# Clinical Impact of Next-Generation Sequencing Multi-Gene Panel Highlighting the Landscape of Germline Alterations in Ovarian Cancer Patients

**DOI:** 10.3390/ijms232415789

**Published:** 2022-12-13

**Authors:** Giorgia Gurioli, Gianluca Tedaldi, Alberto Farolfi, Elisabetta Petracci, Claudia Casanova, Giuseppe Comerci, Rita Danesi, Valentina Arcangeli, Mila Ravegnani, Daniele Calistri, Valentina Zampiga, Ilaria Cangini, Eugenio Fonzi, Alessandra Virga, Davide Tassinari, Marta Rosati, Paola Ulivi, Ugo De Giorgi

**Affiliations:** 1 Biosciences Laboratory, IRCCS Istituto Romagnolo per lo Studio dei Tumori (IRST) “Dino Amadori”, 47014 Meldola, Italy; 2 Department of Medical Oncology, IRCCS Istituto Romagnolo per lo Studio dei Tumori (IRST) “Dino Amadori”, 47014 Meldola, Italy; 3 Biostatistics and Clinical Trials Unit, IRCCS Istituto Romagnolo per lo Studio dei Tumori (IRST) “Dino Amadori”, 47014 Meldola, Italy; 4 Oncology Department, Santa Maria Delle Croci Hospital, 48121 Ravenna, Italy; 5 Department of Obstetrics and Gynaecology, AUSL Romagna, Santa Maria Delle Croci Hospital, 48121 Ravenna, Italy; 6 Romagna Cancer Registry, IRCCS Istituto Romagnolo per lo Studio dei Tumori (IRST) “Dino Amadori”, 47014 Meldola, Italy; 7 Department of Oncology, Ospedale Infermi, 47923 Rimini, Italy

**Keywords:** ovarian cancer, *BRCA1/2*, cancer predisposition, platinum sensitivity, PARP inhibitors, platelets

## Abstract

*BRCA1* and *BRCA2* are the most frequently mutated genes in ovarian cancer (OC) crucial both for the identification of cancer predisposition and therapeutic choices. However, germline variants in other genes could be involved in OC susceptibility. We characterized OC patients to detect mutations in genes other than *BRCA1/2* that could be associated with a high risk of developing OC and permit patients to enter the most appropriate treatment and surveillance program. Next-generation sequencing analysis with a 94-gene panel was performed on germline DNA of 219 OC patients. We identified 34 pathogenic/likely pathogenic variants in *BRCA1/2* and 38 in other 21 genes. The patients with pathogenic/likely pathogenic variants in the non-*BRCA1/2* genes mainly developed OC alone compared to the other groups that also developed breast cancer or other tumors (*p* = 0.001). Clinical correlation analysis showed that the low-risk patients were significantly associated with platinum sensitivity (*p* < 0.001). Regarding PARP inhibitors (PARPi) response, the patients with pathogenic mutations in the non-*BRCA1/2* genes had worse PFS and OS. Moreover, a statistically significantly worse PFS was found for every increase of one thousand platelets before PARPi treatment. To conclude, knowledge about molecular alterations in genes beyond *BRCA1/2* in OC could allow for more personalized diagnostic, predictive, prognostic, and therapeutic strategies for OC patients.

## 1. Introduction

In 2020, ovarian cancer (OC) placed 8th for both incidence and mortality among women [1]. OC is defined as a “silent killer” as it is often diagnosed at advanced stages, resulting in a high mortality rate. About 10–30% of breast and ovarian cancers show familial aggregation and 5–10% of them are considered hereditary, i.e., linked to a germline genetic variant in a cancer predisposition gene [2]. *BRCA1* and *BRCA2* are two of the most frequently mutated genes in high-grade serous OC, which is responsible for the majority of OC deaths [3]. Surgery represents the standard treatment, followed by platinum–taxane chemotherapy. Platinum-resistant OCs recur in nearly 25% of patients within six months and the probability of survival after five years is about 30% [4]. Investigating the *BRCA1/2* mutational status in patients with OC is crucial not only for the identification of familial cancer predisposition, but also to address therapeutic choices. Indeed, patients with germline or somatic variants of *BRCA1* or *BRCA2* have been correlated to a better prognosis and a better response to platinum-based chemotherapy [5,6,7,8]. Moreover, inhibitors of the poly (ADP-ribose) polymerase (PARPi) have been demonstrated to be effective in germline and somatic *BRCA1*- or *BRCA2*-mutated OCs [9,10,11], except for Niraparib that has been shown to be effective regardless of the presence of *BRCA1/2* variants or the homologous recombination deficiency (HRD) status [12].

About 13% of high-grade serous OCs show germline variants in *BRCA1* or *BRCA2* [13,14] and a smaller percentage can be attributed to other germline variants. The *BRCA1* and *BRCA2* genes are the main actors involved in the homologous recombination (HR) DNA repair pathway, together with *ATM*, *BARD1*, *NBN*, and others [15]. Indeed, about 30% of patients with OC present germline and somatic variants in HR genes, of which up to 75% are in the *BRCA1* and *BRCA2* genes [5,15]. Therefore, the detection of new genes determining susceptibility to cancer is an urgent need. To date, other genes have been associated with inherited OC predisposition, particularly *BRIP1*, *RAD51C*, *RAD51D*, *PALB2*, *STK11*, and mismatch repair (MMR) genes, such as *MLH1*, *MSH2*, *MSH6*, and *PMS2*, and others [16,17,18]. Next-generation sequencing (NGS) advent has enabled the analysis of a high number of genes simultaneously with lower costs and a wider access to molecular tests for patients with suspected hereditary cancer or for eligibility for PARPi treatment [19,20,21,22].

In this study, we performed a molecular characterization of patients with OC to identify predisposing genes other than *BRCA1* and *BRCA2* that could permit a better selection of patients with a high risk of developing OC and allowing them to enter the most appropriate treatment and surveillance program.

## 2. Results

### 2.1. Clinicopathological Features

Between January 2014 and December 2018, 219 patients were recruited in this study. All the patients had a diagnosis of OC: 182 patients developed OC alone, 16 developed OC and breast cancer, and 21 patients developed OC and another non-breast-cancer tumor. The median age at diagnosis was 60 years. One hundred sixty-two patients of the 219 patients had high-grade serous OC, 16 had endometrioid OC, and 41 patients presented other histologies. Twelve patients developed G1 OC, 4 patients developed G2 OC, and 188 patients developed G3 OC (data not available for 15 patients). Forty-three patients had stage I/II OC and 164 had stage III/IV OC (data not available for 12 patients).

### 2.2. NGS Analysis

The molecular analysis of the 219 patients showed a mean target coverage of 404 X and a 95.3% mean percentage of the target covered > 50 X.

We observed 42,583 variants in the exonic and splicing regions of 94 genes.

### 2.3. BRCA1/2 Variants

We observed 2501 variants in the *BRCA1/2* genes classified according to the IARC guidelines and online databases as 34 pathogenic/likely pathogenic variants (14 in *BRCA1* and 20 in *BRCA2*), 17 variants of uncertain significance (VUS) (4 in *BRCA1* and 13 in *BRCA2*), and 2450 benign variants. The 34 *BRCA1/2* pathogenic/likely pathogenic variants were present in 34/219 patients (15.5%); in particular, 14/219 (6.4%) had a *BRCA1* mutation (mean age, 55.93 ± 6.4 years) and 20/219 (9.1%) had a *BRCA2* mutation (mean age, 64 ± 7.47 years). The mutation details are shown in Supplementary Table S1. We also found that 17/219 patients (7.8%) harbored a VUS in the *BRCA1/2* genes, of whom two also had a pathogenic variant in *BRCA1*.

### 2.4. Pathogenic Variants in Other Genes

We also analyzed the other 92 genes of the panel, and we observed a total of 40,082 variants that were classified according to the ACMG guidelines in 38 pathogenic/likely pathogenic variants, 4710 VUS, and 35,334 benign variants. The 38 pathogenic/likely pathogenic variants were present in 21 genes in 36/219 patients (16.4%): *PPM1D* (8 variants), *MUTYH* (4), *MITF* (3), *RAD51C* (3), *BRIP1* (2), *ALK* (2), *CHEK2* (2), *PRF1* (1), *PALB2* (1), *FANCD2* (1), *ERCC5* (1), *MLH1* (1), *SBDS* (1), *TP53* (1), *EGFR* (1), *RECQL4* (1), *ERCC2* (1), *MSH2* (1), *ERCC3* (1), *FANCL* (1), *HOXB13* (1).

The mutation details are shown in Supplementary Table S2. Out of the 36 patients, two had pathogenic/likely pathogenic variants in two different genes (patient B184 in *CHEK2* and *EGFR*, and patient B421 in *FANCD2* and *ALK*), while four also had a pathogenic mutation in the *BRCA1/2* genes (one in *BRCA1* and three in *BRCA2*), so these four patients were considered in the *BRCA1/2*-mutated group.

### 2.5. Molecular Subgroups: Clinical and Pathological Comparison

The clinical and pathological information on the 219 patients included in this study is summarized in Table 1.

Our patient cohort was grouped based on gene mutations and the presence of OC alone or OC and additional tumors (Table 2). We observed an association between the type of tumor and the mutational status (*p* < 0.001). In particular, the patients with pathogenic variants in the genes other than *BRCA1/2* mainly developed OC alone compared to the other groups. The patients with OC and breast cancer had predominantly *BRCA1/2* pathogenic mutations, whereas only three out of 21 patients with OC and other tumors had a pathogenic variant. All the three patients who developed OC and breast cancer with a germline *BRCA1* mutation had a breast cancer diagnosis (median age, 42 years) before the onset of OC (median age, 62 years). Again, in the seven patients with a germline *BRCA2* mutation, the onset of breast cancer always preceded OC diagnosis, with a median age of 52 years and 66 years, respectively.

The relationship between the neutrophils, lymphocytes, platelets, neutrophil-to-lymphocyte ratio (NLR), platelet-to-lymphocyte ratio (PLR), and systemic immune inflammation index (SII) before treatment initiation and the mutational status was investigated. We did not observe any significant association (Supplementary Table S3).

### 2.6. Platinum Sensitivity

Firstly, the association between platinum sensitivity and the mutational status was investigated. No statistically significant association between platinum sensitivity and the mutational status was observed (Table 3).

We then analyzed the response to platinum-based chemotherapy of 208 patients (11 patients were undetermined). The OC patients were classified as low-risk if the residual disease did not occur during the primary debulking surgery. The patients with residual disease and/or patients who underwent interval surgery were classified as high-risk. The median time from the last administration of platinum-based chemotherapy and the relapse or death was 24.2 months (IQ range, 10.61–43.50) in 208 patients, 35.8 months (IQ range, 14.98–58.11) in the low-risk group (115 patients) and 15.2 months (IQ range, 6.50–26.41) in the high-risk group (93 patients), *p* < 0.001.

Supplementary Table S4 shows the association between platinum sensitivity and histology.

Data on the pre-treatment inflammatory index (NLR, PLR, SII) levels were available for the 118 patients enrolled. The median NLR value was 246 (IQ range, 171–339), the median PLR value was 200 (IQ range, 140–280), and the median SII was 739 (IQ range, 432–1349). We did not observe any significant correlation between the inflammatory indexes and the mutational status.

### 2.7. PARP Sensitivity

In our case series, 43 patients were treated with maintenance treatment in subsequent lines rather than the first one. Twelve of them presented a pathogenic mutation in *BRCA1/2* (five in *BRCA1* and seven in *BRCA2*), four patients had pathogenic/likely pathogenic variants in four genes other than *BRCA1/2* (*PALB2, ERCC2, ALK*, and *MITF*), and 27 patients had no pathogenic mutations in any of the 94 genes.

The mutational status was analyzed in relation to PARP sensitivity in 43 patients. We observed that the patients without pathogenic variants had a similar outcome compared to the patients with *BRCA1/2* pathogenic variants (HR = 1.15, 95% CI, 0.53–2.48, *p* = 0.715; HR = 0.72, 95% CI, 0.27–1.91, *p* = 0.511, for PFS and OS, respectively). Differently, the patients with pathogenic variants in the genes other than *BRCA1/2* had a significantly worse PFS (HR = 3.56, 95% CI, 1.05–12.04, *p* = 0.042) and a worse OS (HR = 1.38, 95% CI, 0.15–12.13, *p* = 0.772), as shown in Table 4.

The response to PARPi was also evaluated in association with the inflammatory indexes before PARPi treatment initiation for 29 patients. Considering NLR, PLR, and SII as continuous variables, no statistically significant association was found for both PFS and OS. However, for every one standard deviation increase in platelets, a statistically significantly worse PFS was found (HR = 1.52, 95% CI, 1.03–2.26, *p* = 0.037), as shown in Table 5. No association was found in relation to OS (HR = 1.18, 95% CI, 0.62–2.27, *p* = 0.614).

## 3. Discussion

In this study, the molecular characterization of the germline DNA of 219 OC patients was provided through a panel of 94 genes, including the genes involved in the main hereditary cancer syndromes, and the correlation with clinical characteristics was studied. The existing clinical genetic tests for OC are based only on *BRCA1* and *BRCA2* analysis despite new evidence of a higher number of genes eligible for testing [16]. In our cohort selected by the IRST Genetic Counseling service and the Oncology Units of the Area Vasta Romagna (AVR) catchment area, we observed a total of 72 pathogenic/likely pathogenic variants in 70/219 (32%) patients. In particular, 14 variants were found in the *BRCA1* gene, 20 variants in *BRCA2*, and 38 pathogenetic/likely pathogenic variants were found in other 21 genes. The 38 pathogenic/likely pathogenic variants in the genes other than *BRCA1/2* were observed in 36 patients, 32 of whom did not present any pathogenic/likely pathogenic variants in the *BRCA1/2* genes. The most frequently mutated genes in our case series were *PPM1D*, *MUTYH*, *MITF*, *RAD51C*, *BRIP1*, *ALK*, and *CHEK2*. Out of the 36 patients with pathogenic/likely pathogenic variants in the other genes, four also harbored a *BRCA1/2* mutation (one in *BRCA1* and three in *BRCA2*). These four patients did not show particular features in the tumor characteristics or in the therapeutic response. Consequently, they were included in the *BRCA1/2*-positive group, presuming a stronger effect of *BRCA1/2* mutations on the phenotype.

*BRCA1* and *BRCA2* are part of the BRCA–Fanconi anemia pathway, and other Fanconi genes, such as *BRIP1* and *RAD51C*, have also been associated with an inherited risk of OC [23,24,25]. *PPM1D* variants have been associated with predisposition to breast and ovarian cancers [26,27] along with *MUTYH* [28,29] and *CHEK2* [29]. Moreover, mutations in the mismatch repair genes that cause Lynch syndrome (*MLH1*, *MSH2, MSH6*, and *PMS2*) also confer a risk for OC [30,31]. In our case series, we detected a likely pathogenic variant in the *MLH1* gene in a patient negative for *BRCA1/2* mutations and a likely pathogenic variant in the *MSH2* gene in a patient with a pathogenic mutation in the *BRCA2* gene. Other authors also underlined that germline sequencing of *BRCA1* and *BRCA2* should be performed in the context of a multigene panel that also includes *RAD51C*, *RAD51D*, *BRIP1*, *MLH1*, *MSH2*, *MSH6*, *PMS2*, and *PALB2* [32]. These data highlighted that these mutations are associated with a higher risk of OC development, so it is noteworthy to introduce a multigene panel in standard genetic analysis protocols for patients with suspected hereditary OC.

The patients enrolled in this study were selected on the basis of OC diagnosis in order to identify the patients eligible for PARP inhibitors treatment. Consequently, we do not have information on the family history of cancer for the majority of them. However, all the *BRCA1/2*-positive patients and the ones with pathogenic/likely pathogenic variants in the genes for which clinical guidelines are available (e.g., *TP53*, *MSH2*, *MLH1*) were referred to appropriate genetic counseling with the reconstruction of the family history of cancer and the extension of the genetic test to the consenting relatives.

Our molecular findings were then analyzed in relation to clinical characteristics. Our cohort of patients was divided into molecular subgroups, and we showed that patients with pathogenic/likely pathogenic variants in the genes other than *BRCA1/2* developed mainly OC alone, suggesting that these genes could be specifically related to OC predisposition. Interestingly, all the patients with a germline *BRCA1* or *BRCA2* mutation and a second malignancy first developed a breast cancer and thereafter an OC. This observation highlights the need for a strict follow-up in order to identify OC early and/or to discuss with the patient risk-reducing salpingo-oophorectomy. This procedure was also demonstrated to reduce the risk of breast cancer in the immediate 5 years after surgery and in the longer term, especially in younger women [33,34].

Although most patients with OC initially respond to platinum-based chemotherapy, about 20% of women experience disease progression ≤ 6 months after the last cycle of a platinum-based regimen (platinum-resistant or platinum-refractory) [35]. Many efforts have been made over the years to develop predictive biomarkers of platinum sensitivity [36].

We found that patients without residual disease after the primary debulking surgery (low-risk patients) had a significantly longer median time from the last administration of platinum-based chemotherapy and the relapse or death (35.8 months) than high-risk patients (15.2 months) (*p* < 0.001). We also hypothesized a better clinical outcome in patients with the DNA damage response genes altered because of a worse platinum-induced DNA interstrand crosslinks repair capability [37].

A subset of 43 patients was treated with PARPi as maintenance treatment after platinum chemotherapy. We observed that the patients with pathogenic mutations in the genes other than *BRCA1/2* had significantly worse PFS and OS compared to the patients with a pathogenic mutation in the *BRCA1/2* genes, suggesting that this may be associated with specific biological mechanisms. However, due to the small number of cases, we could not speculate about it.

On the other hand, the patients without pathogenic/likely pathogenic variants had a similar outcome to the patients with a pathogenic variant in the *BRCA1/2* genes, confirming literature data [38].

Regarding inflammation indexes correlation, we previously demonstrated that inflammatory indexes (NLR and SII) are independent prognostic factors in recurrent platinum-sensitive OC patients [36]. In this analysis, only platelets were correlated with PFS, demonstrating their important role in OC not only as a poor prognostic factor [39], but also as possible predictive factor of response to PARPi. However, a validation of these easy biomarkers in a larger case series is warranted.

One of the aims of this study was to identify new genes involved in the predisposition to OC. For this reason, we chose to analyze a panel of 94 genes involved in different forms of hereditary cancer and include all the pathogenic/likely pathogenic variants in the analysis. However, this approach has some limitations. Indeed, literature statistics describe only a minor fraction of OC cases attributable to genes other than *BRCA1/2* [23,40]. In particular, the eight *PPM1D* variants detected could be treatment-related mosaic somatic mutations, as suggested by other authors [41], but we considered them as real germline variants since one of them was present in three different patients, and among the five remaining variants, three were already reported in the literature and one of the two unreported variants was present in a patient with a *BRCA2* pathogenic variant. Moreover, the VAF (variant allele frequency) was compatible with a germline nature of the mutations (~0.5). Regarding the *ALK* gene, one of the two mutations was present in a patient with also a *FANCD2* pathogenic variant. Moreover, *ALK* and *MITF* germline alterations were associated with neuroblastoma and melanoma risk, respectively, but this finding is intriguing for possible links with other cancers such as OC. Two *CHEK2* pathogenic variants were found in patients with high-grade serous ovarian cancer. The *CHEK2* gene is mainly linked to ovarian cystadenomas, borderline ovarian tumors, and low-grade invasive cancers [42], but at the same time this gene is involved in DNA damage response and is associated with a moderate risk of breast cancer [18] and also a risk of other tumors [43], such as prostate [44,45,46], colorectal [47], and gastric cancers [48]. Regarding the *MUTYH* gene, only biallelic mutations are well-known for their association with cancer risk [49], but an increased risk for cancer has also been reported for monoallelic carriers since the conjunction of a germline mutation with a somatic mutation may also contribute to the development of OC [28]. For these reasons, we decided to include all these patients in the group of carriers of pathogenic/likely pathogenic variants in the non-*BRCA1/2* genes.

## 4. Materials and Methods

### 4.1. Ethics Statement

The study was performed in accordance with the Good Clinical Practice and the Declaration of Helsinki and approved by the AVR Ethics Committee (protocol No. 6326/2020). All the patients enrolled in the study signed informed consent forms for genetic analyses and for the use of the results for research purposes.

### 4.2. Patients and Samples

Patients with a diagnosis of OC referring to the IRST Genetic Counseling service or to the Oncology Units of the AVR catchment area in 2014–2018 were included in this study. A total of 219 patients were recruited and tested for a multi-gene panel, including the search for *BRCA1/2* alterations for possible treatment with PARPi.

Peripheral blood of patients was collected and stored at −80 °C for subsequent molecular analyses. Genomic DNA was extracted with a QIAamp DNA mini kit (Qiagen, Hilden, Germany) and quantified using a Qubit dsDNA BR Assay kit (Thermo Fisher Scientific, Waltham, MA, USA).

### 4.3. Next-Generation Sequencing (NGS) Analyses

Sequencing libraries were generated using 50 ng of genomic DNA. Libraries were enriched for the regions of interest with the Trusight Cancer panel (Illumina, San Diego, CA, USA), including the coding regions and flanking introns of 94 genes involved in hereditary cancer (Supplementary Table S5). Sequencing was performed using the MiSeq platform (Illumina) with MiSeq Reagent Kit v2 configured for 2 × 150 cycles according to the manufacturer’s instructions as previously described [50,51].

### 4.4. Data Analysis and Variant Calling

Paired-end sequencing reads were aligned to the reference human genome (UCSC hg19) with Burrows–Wheeler algorithm v0.7.15-r1140 [52]. The sequences around insertions and deletions (indels) were realigned locally with GATK v3.6-0 [53]. Then, Picard MarkDuplicates v2.6.0 (http://broadinstitute.github.io/picard/, accessed on 16 October 2022) was used to remove duplicate read-pairs artifacts arising during PCR amplification or sequencing. The data then underwent base quality score recalibration (BQSR) to ensure good call quality and reduce the number of false positives (again, with GATK). Variant calling was separately performed with GATK UnifiedGenotyper and freebayes v1.0.2-58 [54]; then, the resulting VCF files were merged with GATK CombineVariants. ANNOVAR v2016-02-01 was used for genomic and functional annotations of the detected variants [55], while coverage statistics were computed with the DepthOfCoverage utility of GATK and the downstream custom bash/R scripts. The resulting annotated list of variants was filtered for variants present in exonic regions or in the 20 bases flanking each exon.

### 4.5. Additional BRCA1/2 Analyses

*BRCA1/2* regions covering < 50 X were amplified by standard PCR and sequenced using a Big Dye Terminator v.3.1 cycle sequencing kit (Thermo Fisher Scientific) on an ABI-3130 Genetic analyzer (Applied Biosystems, Waltham, MA, USA). Multiplex ligation-dependent probe amplification (MLPA) analysis with BRCA1-P002 and BRCA2-P045 kits (MRC Holland, Amsterdam, The Netherlands) was performed to identify gross deletions/insertions not detectable by sequencing. The MLPA results were analyzed using the Coffalyser software (MRC Holland).

### 4.6. Variants Classification

Genetic variants were classified into five classes according to the IARC recommendations [56]. The *BRCA1/2* variants classification was performed by consulting ClinVar [57] and the main *BRCA1/2* mutation databases, such as BRCA Exchange, BRCA Share, and LOVD [58,59,60]. Sequence variants in the other 92 genes were classified using ClinVar [57] and dbSNP [61]. The variants absent in any of these databases were classified using VarSome [62] in accordance with the guidelines of the American College of Medical Genetics [63].

### 4.7. Inflammatory Indexes

Information on the neutrophil, lymphocyte, and platelet counts from blood tests carried out at baseline was collected. The SII was calculated as the (platelet count × neutrophil count)/lymphocyte count, the NLR was obtained by dividing the absolute neutrophil count by the absolute lymphocyte count, and the PLR was calculated as the ratio of the absolute platelet count to the absolute lymphocyte count [64]. The inflammatory indexes value was multiplied by 100. The median value of the SII, NLR, and PLR was considered.

### 4.8. Statistical Analysis

The data were summarized as the mean ± standard deviation (SD) or median, interquartile (IQ) range and the minimum and maximum value, as appropriate, for the continuous variables and through natural frequencies and percentages for the categorical ones. The associations between the categorical variables were tested by Pearson’s χ^2^ test or Fisher’s exact test, as appropriate, whereas those between a continuous variable and a categorical one were tested by means of Student’s t-test or the F-test or analogous nonparametric tests, when appropriate.

Platinum sensitivity was defined as the time in months from the date of the end of platinum-based chemotherapy until the date of relapse or death from any cause, whichever occurred first. The alive patients without relapse were censored at the time of the last follow-up.

The prognosis of the patients treated with PARPi was investigated in terms of progression-free survival (PFS) defined as the time in months from the date of inhibitor initiation until disease progression or death from any cause, whichever occurred first, and overall survival (OS) was defined as the time in months from the date of inhibitor initiation until death from any cause. The patients were censored at the date of the last follow-up update.

The time-to-event outcomes were analyzed by means of the Cox proportional hazards model; the effect of the biological and clinical covariates was reported in terms of hazard ratios (HRs) and the corresponding 95% confidence intervals (CIs).

All the statistical analyses were performed using the STATA 15.0 software (College Station, TX, USA).

## 5. Conclusions

To conclude, knowledge about molecular alterations in the genes beyond *BRCA1/2* in OC could allow for more personalized diagnostic, predictive, prognostic, and therapeutic strategies for patients without forgetting the clinical implications for her family members.

## Figures and Tables

**Table 1:** Patient characteristics according to the mutational status.

	Patients with the *BRCA1* Pathogenic Variants (*n* = 14)	Patients with the *BRCA2* Pathogenic Variants (*n* = 20)	Patients with the Other Pathogenic Variants (*n* = 32)	Patients without Pathogenic Variants (*n* = 153)	Total (*n* = 219)	*p-*Value
**Age at diagnosis**						0.083
Mean ± SD	55.93 ± 6.40	64.00 ± 7.47	62.52 ± 14.26	59.55 ± 11.34	60.10 ± 11.30	
Missing	–	1	5	6	12	
**Histology**						0.497
High-grade serous	11 (78.57)	19 (95.00)	23 (71.88)	109 (71.24)	162 (73.97)	
Endometrioid	1 (7.14)	0	2 (6.25)	13 (8.50)	16 (7.31)	
Other histology	2 (14.29)	1 (5.00)	7 (21.88)	31 (20.26)	41 (18.72)	
**Grade**						0.721
G1	1 (7.14)	0	3 (11.11)	8 (5.56)	12 (5.88)	
G2	0	0	0	4 (2.78)	4 (1.96)	
G3	13 (92.86)	19 (100.00)	24 (88.89)	132 (91.67)	188 (92.16)	
Missing	–	1	5	9	15	
**Stage**						0.760
I/II	3 (21.43)	2 (10.53)	6 (22.22)	32 (21.77)	43 (20.77)	
III/IV	11 (78.57)	17 (89.47)	21 (77.78)	115 (78.23)	164 (79.23)	
Missing	–	1	5	6	12	
**Ascites**						0.766
No	8 (61.54)	12 (63.16)	19 (70.37)	87 (59.59)	126 (61.46)	
Yes	5 (38.46)	7 (36.84)	8 (29.63)	59 (40.41)	79 (38.54)	
Missing	1	1	5	7	14	
**Visceral metastases**						0.491
No	5 (83.33)	11 (84.62)	12 (93.31)	61 (92.42)	89 (90.82)	
Yes	1 (16.67)	2 (15.38)	1 (7.69)	5 (7.58)	9 (7.18)	
Missing	8	7	19	87	121	
**Risk**						0.925
Low	7 (50.00)	10 (52.63)	14 (51.85)	84 (56.76)	115 (55.29)	
High	7 (50.00)	9 (47.37)	13 (48.15)	64 (43.24)	93 (44.71)	
Missing	–	1	5	5	11	

**Table 2:** Spectrum of tumors according to the mutational status.

	Patients with the *BRCA1* Pathogenic Variants (*n* = 14)	Patients with the *BRCA2* Pathogenic Variants (*n* = 20)	Patients with the Other Pathogenic Variants (*n* = 32)	Patients without Pathogenic Variants (*n* = 153)	Total (*n* = 219)	*p-*Value
**Ovarian cancer**	11 (78.57)	12 (60.00)	30 (93.75)	129 (84.31)	182	<0.001
**Ovarian and breast cancer**	3 (21.43)	7 (35.00)	0	6 (3.92)	16	
**Ovarian cancer and other tumors**	0	1 (5.00)	2 (6.25)	18 (11.76)	21	

**Table 3:** Platinum sensitivity and mutational status.

	Patients with the *BRCA1* Pathogenic Variants (*n* = 14)	Patients with the *BRCA2* Pathogenic Variants (*n* = 20)	Patients with the Other Pathogenic Variants (*n* = 32)	Patients without Pathogenic Variants (*n* = 153)	Total (*n* = 219)	*p-*Value
**Platinum sensitivity, months ^§^**						0.167
Median (IQ range)	15.76 (7.84–37.5)	42.51 (15.37–52.1)	21.77 (8.8–33.76)	23.95 (9.66–43.00)	23.71 (10.05–43.25)	
Minimum–maximum	5.35–108.31	4.27–124.74	0–118.86	0.16–329.4	0–329.4	
Missing	2	3	8	13	26	

**^§^**Time from the date of the end of platinum-based chemotherapy until the date of relapse or death from any cause.

**Table 4:** Progression-free survival (PFS) and overall survival (OS) according to the mutational status.

	PFS		OS	
	HR (95% CI)	*p-*Value	HR (95% CI)	*p-*Value
**Mutational status**				
Patients with the *BRCA1/2* pathogenic variants	1		1	
Patients with the other pathogenic variants	3.56 (1.05–12.04)	0.042	1.38 (0.15–12.13)	0.772
Patients without pathogenic variants	1.15 (0.53–2.48)	0.715	0.72 (0.27–1.91)	0.511

**Table 5:** Results from univariate Cox analysis between the inflammatory indexes and PFS.

	HR (95% CI)	*p-*Value
**Neutrophils**	0.99 (0.66–1.50)	0.981
**Lymphocytes**	1.02 (0.69–1.50)	0.922
**Platelets**	1.52 (1.03–2.26)	0.037
**NLR**	0.93 (0.64–1.36)	0.702
**PLR**	1.51 (0.97–2.34)	0.067
**SII**	1.31 (0.88–1.96)	0.184

## Data Availability

The data presented in this study are available on request from the corresponding author.

## Supplementary Materials

The following supporting information can be downloaded at https://www.mdpi.com/article/10.3390/ijms232415789/s1.
