# Supplementary material for: Clinical Impact of Next-Generation Sequencing Multi-Gene Panel Highlighting the Landscape of Germline Alterations in Ovarian Cancer Patients
Source: Int J Mol Sci. 2022 Dec 13;23(24):15789. doi: 10.3390/ijms232415789 (PMC9779064; doi:10.3390/ijms232415789)
Supplement: Supplementary file 1 [file ijms-23-15789-s001.zip › ijms-2007273-SI.pdf]

Supplementary Table S1. List of the 34 heterozygous *BRCA1/2* pathogenic/likely-pathogenic variants identified in the case series.

Patient ID	1st cancer	Age at onset	2nd cancer	Age at onset	Gene	Exon	DNA (HGVS)*	Protein (HGVS)**	Variant type	IARC class	dbSNP	ClinVar/Other databases
A125	BC	48	OC	50	*BRCA2*	23	c.9097dupA	p.Thr3033AsnfsTer11	frameshift insertion	5	rs397507419	pathogenic
A284	OC	49	-	-	*BRCA1*	20	c.5266dupC	p.Gln1756ProfsTer74	frameshift insertion	5	rs80357906	pathogenic
A643	BC	66	OC	70	*BRCA2*	11	c.3897_3901del	p.Glu1299AspfsTer7	frameshift deletion	4	-	-
A793	BC	54	OC	67	*BRCA2*	11	c.3847_3848del	p.Val1283LysfsTer2	frameshift deletion	5	rs80359405	pathogenic
A835	BC	40	OC	61	*BRCA1*	14	c.4484G>T	p.Arg1495Met	missense variant	5	rs80357389	pathogenic
A882	OC	55	-	-	*BRCA1*	11	c.4035delC	p.Glu1346LysfsTer20	frameshift deletion	5	rs80357711	pathogenic
A884	BC	52	OC	60	*BRCA2*	11	c.3743_3746del	p.Ser1248ArgfsTer10	frameshift deletion	5	rs80359403	pathogenic
A891	OC	64	-	-	*BRCA1*	11	c.1513A>T	p.Lys505Ter	nonsense variant	5	rs397508877	pathogenic
A899	OC	61	BC	66	*BRCA2*	16	c.7618-2A>G	p.?	splicing variant	5	rs886040940	pathogenic
A922	BC	42	OC	66	*BRCA2*	11	c.6468_6469del	p.Gln2157IlefsTer18	frameshift deletion	5	rs80359596	pathogenic
A938	BC	46	OC	52	*BRCA1*	8	c.529delT	p.Ser177LeufsTer57	frameshift deletion	5	rs80357758	pathogenic
B160	OC	66	-	-	*BRCA2*	11	c.4889C>G	p.Ser1630Ter	nonsense variant	5	rs80358711	pathogenic
B165	OC	74	-	-	*BRCA2*	14	c.7180A>T	p.Arg2394Ter	nonsense variant	5	rs80358946	pathogenic
B166	BC	71	OC	71	*BRCA1*	11	c.3748G>T	p.Glu1250Ter	nonsense variant	5	rs28897686	pathogenic
B215	OC	66	RCC	65	*BRCA2*	11	c.5868dupT	p.Ile1957TyrfsTer3	frameshift insertion	4	-	-
B220	OC	51	-	-	*BRCA2*	13	c.6998dupT	p.Pro2334ThrfsTer6	frameshift insertion	5	rs754611265	pathogenic
B245	OC	57	-	-	*BRCA1*	11	c.3700_3704del	p.Val1234GlnfsTer8	frameshift deletion	5	rs80357609	pathogenic
B270	OC	56	-	-	*BRCA1*	10	c.615dupA	p.Gln206ThrfsTer10	frameshift insertion	4	rs1567803215	VUS/likely-pathogenic
B294	OC	64	-	-	*BRCA2*	11	c.3046G>T	p.Glu1016Ter	nonsense variant	5	rs748508287	pathogenic
B295	OC	57	-	-	*BRCA2*	10	c.1813delA	p.Ile605TyrfsTer9	frameshift deletion	5	rs80359306	pathogenic
B319	OC	53	-	-	*BRCA1*	24	c.5503C>T	p.Arg1835Ter	nonsense variant	5	rs41293465	pathogenic
B336	OC	63	-	-	*BRCA2*	11	c.6037A>T	p.Lys2013Ter	nonsense variant	5	rs80358840	pathogenic

B351	OC	65	-	-	*BRCA2*	11	c.4284dupT	p.Gln1429SerfsTer9	frameshift insertion	5	rs80359439	pathogenic
B359	OC	49	-	-	*BRCA1*	14	c.4484G>T	p.Arg1495Met	missense variant	5	rs80357389	pathogenic
B363	OC	49	-	-	*BRCA1*	24	c.5468-1G>A	p.?	splicing variant	5	rs80358048	pathogenic
B365	OC	54	-	-	*BRCA1*	11	c.850C>T	p.Gln284Ter	nonsense variant	5	rs397509330	pathogenic
B372	OC	52	-	-	*BRCA1*	11	c.850C>T	p.Gln284Ter	nonsense variant	5	rs397509330	pathogenic
B404	OC	76	-	-	*BRCA2*	11	c.2905C>T	p.Gln969Ter	nonsense variant	5	rs886038080	pathogenic
B409	BC	35	OC	58	*BRCA2*	11	c.2684delC	p.Ala895ValfsTer9	frameshift deletion	5	rs80359342	pathogenic
B413	OC	58	-	-	*BRCA2*	11	c.4284dupT	p.Gln1429SerfsTer9	frameshift insertion	5	rs80359439	pathogenic
B418	OC	61	-	-	*BRCA1*	14	c.4484G>T	p.Arg1495Met	missense variant	5	rs80357389	pathogenic
B465	OC	69	-	-	*BRCA2*	11	c.2684delC	p.Ala895ValfsTer9	frameshift deletion	5	rs80359342	pathogenic
B519	OC	77	-	-	*BRCA2*	27	c.9871del	p.Ser3291LeufsTer22	frameshift deletion	5	rs886040854	pathogenic
B682	OC	64	-	-	*BRCA2*	11	c.3847_3848del	p.Val1283LysfsTer2	frameshift deletion	5	rs80359405	pathogenic

OC: ovarian cancer
BC: breast cancer
RCC: renal cell carcinoma
VUS: variant of uncertain significance

* *BRCA1* transcript: NM_007294.4; *BRCA2* transcript: NM_000059.4
** BRCA1 protein: NP_009225.1; BRCA2 protein: NP_000050.3

Supplementary Table S2. List of the 38 heterozygous pathogenic/likely-pathogenic variants in genes other than *BRCA1/2* identified in the case series.

Patient ID	1st cancer	Age at onset	2nd cancer	Age at onset	*BRCA1/2* status	Gene	Transcript	Exon	DNA (HGVS)	Protein (HGVS)	Variant type	IARC class	dbSNP	ClinVar
A284	OC	49	-	-	*BRCA1+*	*ERCC3*	NM_000122	11	c.1757delA	p.Gln586ArgfsTer25	frameshift deletion	5	rs753182861	pathogenic
A893	OC	52	-	-	wt	*PPM1D*	NM_003620	6	c.1535dupA	p.Asn512LysfsTer16	frameshift insertion	4	rs763475304	-
A906	OC	50	-	-	wt	*SBDS*	NM_016038	2	c.258+2T>C	p.?	splicing variant	5	rs113993993	pathogenic
A912	OC	74	-	-	wt	*PPM1D*	NM_003620	6	c.1426G>T	p.Glu476Ter	nonsense variant	4	rs1296018768	-
A913	OC	64	-	-	wt	*PPM1D*	NM_003620	6	c.1654C>T	p.Arg552Ter	nonsense variant	5	rs779070661	pathogenic
A916	NET	69	OC	73	wt	*BRIP1*	NM_032043	9	c.1201_1204dup	p.Ala402ValfsTer21	frameshift insertion	5	rs730881647	pathogenic
A917	OC	59	-	-	wt	*PPM1D*	NM_003620	6	c.1273delG	p.Asp425IlefsTer6	frameshift deletion	4	-	-
A918	OC	54	-	-	wt	*BRIP1*	NM_032043	8	c.1018_1019insCT	p.Leu340ProfsTer9	frameshift insertion	5	rs878855134	pathogenic
A939	OC	75	-	-	wt	*TP53*	NM_000546	8	c.817C>T	p.Arg273Cys	missense variant	5	rs121913343	pathogenic
A944	OC	65	-	-	wt	*PPM1D*	NM_003620	6	c.1654C>T	p.Arg552Ter	nonsense variant	5	rs779070661	pathogenic
B144	OC	47	-	-	wt	*MUTYH*	NM_012222	12	c.1162C>T	p.Gln388Ter	nonsense variant	5	rs587783057	pathogenic
B167	OC	54	-	-	wt	*MUTYH*	NM_012222	13	c.1178G>A	p.Gly393Asp	missense variant	5	rs36053993	pathogenic
B184	OC	77	-	-	wt	*EGFR*	NM_005228	7	c.844G>T	p.Glu282Ter	nonsense variant	4	-	-
						CHEK2	NM_007194	11	c.1232G>A	p.Trp411Ter	nonsense variant	5	rs371418985	pathogenic
B204	OC	79	-	-	wt	*PPM1D*	NM_003620	6	c.1281G>A	p.Trp427Ter	nonsense variant	5	rs1064797099	pathogenic
B205	OC	39	-	-	wt	*ALK*	NM_004304	16	c.2782dupT	p.Cys928LeufsTer20	frameshift insertion	4	rs1218092221	-
B220	OC	51	-	-	*BRCA2+*	*HOXB13*	NM_006361	1	c.251G>A	p.Gly84Glu	missense variant	4	rs138213197	conflicting
B243	OC	75	-	-	wt	*RECQL4*	NM_004260	15	c.2300delT	p.Val767GlyfsTer76	frameshift deletion	4	rs752895803	-
B303	OC	56	-	-	wt	*RAD51C*	NM_058216	7	c.905-2_905-1del	p.?	splicing variant	5	rs587781995	pathogenic
B330	OC	70	-	-	wt	*MITF*	NM_000248	9	c.952G>A	p.Glu318Lys	missense variant	5	rs149617956	pathogenic
B336	OC	63	-	-	*BRCA2+*	*PPM1D*	NM_003620	6	c.1465delT	p.Ser489LeufsTer2	frameshift deletion	4	-	-
B357	OC	85	-	-	wt	*PRF1*	NM_005041	2	c.160C>T	p.Arg54Cys	missense variant	5	rs200430442	pathogenic
B391	OC	46	-	-	wt	*MITF*	NM_000248	9	c.952G>A	p.Glu318Lys	missense variant	5	rs149617956	pathogenic

B406	OC	69	-	-	wt	*PALB2*	NM_024675	4	c.1140_1143del	p.Ser380ArgfsTer43	frameshift deletion	5	rs1257545151	pathogenic
B419	OC	73	-	-	wt	*ERCC2*	NM_000400	21	c.2005_2006del	p.Arg669GlyfsTer104	frameshift deletion	4	rs757535186	-
B421	OC	40	-	-	wt	*ALK*	NM_004304	16	c.2782dupT	p.Cys928LeufsTer20	frameshift insertion	4	rs1218092221	-
						FANCD2	NM_033084	35	c.3541C>T	p.Gln1181Ter	nonsense variant	5	-	pathogenic
B426	OC	78	-	-	wt	*RAD51C*	NM_058216	1	c.93delG	p.Phe32SerfsTer8	frameshift deletion	5	rs730881942	pathogenic
B458	OC	76	-	-	wt	*ERCC5*	NM_000123	15	c.3285_3294del	p.Ser1096AspfsTer12	frameshift deletion	4	-	-
B476	TC	26	OC	45	wt	*MUTYH*	NM_012222	7	c.527A>G	p.Tyr176Cys	missense variant	5	rs34612342	pathogenic
B513	OC	54	-	-	wt	*RAD51C*	NM_058216	7	c.905-2_905-1del	p.?	splicing variant	5	rs587781995	pathogenic
B519	OC	77	-	-	*BRCA2*+	*MSH2*	NM_000251	5	c.942+2delT	p.?	splicing variant	4	rs587779194	likely-pathogenic
B542	OC	82	-	-	wt	*PPM1D*	NM_003620	6	c.1654C>T	p.Arg552Ter	nonsense variant	5	rs779070661	pathogenic
B571	OC	51	-	-	wt	*FANCL*	NM_018062	14	c.1096_1099dup	p.Thr367AsnfsTer13	frameshift insertion	4	rs759217526	conflicting
B612	OC	78	-	-	wt	*CHEK2*	NM_007194	4	c.514dupA	p.Thr172AsnfsTer14	frameshift insertion	5	rs1601823546	pathogenic
B618	OC	68	-	-	wt	*MUTYH*	NM_012222	13	c.1178G>A	p.Gly393Asp	missense variant	5	rs36053993	pathogenic
B693	OC	43	-	-	wt	*MLH1*	NM_000249	12	c.1039-1G>C	p.?	splicing variant	4	rs267607819	likely-pathogenic
B697	OC	54	-	-	wt	*MITF*	NM_000248	9	c.952G>A	p.Glu318Lys	missense variant	5	rs149617956	pathogenic

OC: ovarian cancer
NET: neuroendocrine tumor
TC: thyroid cancer

Supplementary Table S3. Levels of neutrophils, lymphocytes, platelets, NLR, PLR, SII before treatment initiation and mutational status.

	BRCA1+ (n=14)	*BRCA2*+ (n=20)	Other genes (n=32)	wt (n=153)	Total (n=219)	*P*
**Neutrophil counts**						0.347
Median<br>[IQ range]	3310<br>[2230 - 5010]	4200<br>[2950 - 5730]	3510<br>[2390 - 4945]	4150<br>[2880 - 5620]	4115<br>[2770 - 5440]	
Min - max	1430 - 11300	2560 – 12970	1600 – 14300	1960 - 11450	1430 - 14300	
missing	4	9	12	64	89	
**Lymphocyte counts**						0.261
Median<br>[IQ range]	1350<br>[945 - 1855]	1480<br>[1280 - 1660]	1530<br>[1320 - 1950]	1645<br>[1365 - 1995]	1585<br>[1330 - 1920]	
Min - max	178 - 2120	690 – 2150	1010 - 3760	260 - 3200	178 - 3760	
missing	6	9	13	73	101	
**Platelet counts**						0.986
Median<br>[IQ range]	339<br>[172 - 403]	331<br>[231 - 488]	325.5<br>[276 – 391.5]	306<br>[255 - 404]	319<br>[256 - 410]	
Min - max	133 - 927	199 - 569	96 - 693	176 - 853	96 - 927	
missing	4	9	12	64	89	
**NLR**						0.156
Median<br>[IQ range]	3.22<br>[2.12 – 5.20]	2.82<br>[1.87 – 4.50]	2.03<br>[1.51 – 3.28]	2.52<br>[1.66 – 3.42]	2.46<br>[1.71 – 3.39]	
Min - max	1.72 – 8.03	1.63 – 13.23	0.75 – 10.51	0.92 – 15.16	0.75 – 15.16	
missing	6	9	13	73	101	
**PLR**						0.360
Median<br>[IQ range]	0.30<br>[0.16 – 0.43]	0.26<br>[0.16 – 0.31]	0.19<br>[0.14 – 0.28]	0.19<br>[0.14 – 0.24]	0.20<br>[0.14 – 0.28]	
Min - max	0.10 – 0.74	0.13 – 0.70	0.05 – 0.58	0.07 – 0.99	0.05 – 0.99	
missing	6	9	13	73	101	
**SII**						0.437
Median<br>[IQ range]	970.33<br>[640.57 – 1109.37]	1029<br>[441.69 – 2077.55]	540.79<br>[357.45 – 1431.96]	774.97<br>[429.32 – 1315.99]	738.55<br>[432.34 – 1348.58]	
Min - max	133 - 927	408.86 – 4380.68	216.12 – 3701.18	197.75 – 7488.41	197.75 – 7488.41	
missing	6	9	13	73	101	
**Dichotomized NLR, n (%)**						0.067
< 2.46	2 (25.00)	4 (36.36)	14 (73.68)	39 (48.75)	59 (50.00)	

≥ 2.46	6 (75.00)	7 (63.64)	5 (26.32)	41 (51.25)	59 (50.00)	
missing	6	9	13	73	101	
**Dichotomized PLR, n (%)**						0.864
< 0.20	3 (37.50)	5 (45.45)	10 (52.63)	42 (52.50)	60 (50.85)	
≥ 0.20	5 (62.50)	6 (54.55)	9 (47.37)	38 (47.50)	58 (49.15)	
missing	6	9	13	73	101	
**Dichotomized SII, n (%)**						0.268
< 738.54	3 (37.50)	4 (36.36)	13 (68.42)	39 (48.75)	59 (50.00)	
≥ 738.54	5 (62.50)	7 (63.64)	6 (31.58)	41 (51.25)	59 (50.00)	
missing	6	9	13	73	101	
**Dichotomized platelets, n (%)**						0.361
≤ 450	8 (80.00)	7 (63.64)	18 (90.00)	73 (82.02)	106 (81.54)	
> 450	2 (20.00)	4 (36.36)	2 (10.00)	16 (17.98)	24 (18.46)	
missing	4	9	12	63	89	

NLR: neutrophil-to-lymphocyte ratio

PLR: platelet-to-lymphocyte ratio

SII: systemic immune-inflammation index

Supplementary Table S4. Association between platinum sensitivity and histology in our case series.

	Serous high-grade (n=162)	Endometrioid (n=16)	Other (n=41)	Total (n=219)	*P*
**Platinum sensitivity, months**§					0.210
Median<br>[IQ range]	22.39<br>[10.40 – 42.82]	19.09<br>[8.71 – 38.3]	37.73<br>[14.85 – 48.52]	24.15<br>[10.61 – 43.50]	
Min - max	0 – 124.74	0.16 – 128.81	0.62 – 329.4	0 – 329.4	
missing	10	1	15	26	

§ Time from the date of the end of platinum-based chemotherapy until the date of relapse or death from any cause.

Supplementary Table S5. List of the 94 genes included in the Trusight Cancer panel

Genes									
AIP	*ALK*	*APC*	*ATM*	*BAP1*	*BLM*	*BMPR1A*	*BRCA1*	*BRCA2*	*BRIP1*
BUB1B	*CDC73*	*CDH1*	*CDK4*	*CDKN1C*	*CDKN2A*	*CEBPA*	*CEP57*	*CHEK2*	*CYLD*
DDB2	*DICER1*	*DIS3L2*	*EGFR*	*EPCAM*	*ERCC2*	*ERCC3*	*ERCC4*	*ERCC5*	*EXT1*
EXT2	*EZH2*	*FANCA*	*FANCB*	*FANCC*	*FANCD2*	*FANCE*	*FANCF*	*FANCG*	*FANCI*
FANCL	*FANCM*	*FH*	*FLCN*	*GATA2*	*GPC3*	*HNF1A*	*HRAS*	*KIT*	*MAX*
MEN1	*MET*	*MLH1*	*MSH2*	*MSH6*	*MUTYH*	*NBN*	*NF1*	*NF2*	*NSD1*
PALB2	*PHOX2B*	*PMS1*	*PMS2*	*PRF1*	*PRKAR1A*	*PTCH1*	*PTEN*	*RAD51C*	*RAD51D*
RB1	*RECQL4*	*RET*	*RHBDF2*	*RUNX1*	*SBDS*	*SDHAF2*	*SDHB*	*SDHC*	*SDHD*
SLX4	*SMAD4*	*SMARCB1*	*STK11*	*SUFU*	*TMEM127*	*TP53*	*TSC1*	*TSC2*	*VHL*
WRN	*WT1*	*XPA*	*XPC*						
